# Fucoidan Protects against Acute Sulfoxaflor-Induced Hematological/Biochemical Alterations and Oxidative Stress in Male Mice

**DOI:** 10.3390/ph15010016

**Published:** 2021-12-24

**Authors:** Petek Piner Benli, Merve Kaya, Yusuf Kenan Dağlıoğlu

**Affiliations:** 1 Department of Veterinary Pharmacology and Toxicology, Faculty of Ceyhan Veterinary Medicine, Cukurova University, 01330 Adana, Turkey; 2 Department of Biotechnology, Institute of Natural and Applied Sciences, Cukurova University, 01330 Adana, Turkey; kkayamerve46@gmail.com; 3 Department of Microbiology, Faculty of Medicine, Kırsehir Ahi Evran University, 40100 Kırsehir, Turkey; kenan.daglioglu@ahievran.edu.tr

**Keywords:** fucoidan, sulfoxaflor toxicity, hematological parameter, biochemical parameters, oxidative stress, mice

## Abstract

Fucoidan is a sulfated polysaccharide which can be found among a number of macroalgea species. It has a broad spectrum of biological activities including anti-oxidant, anti-tumor, immunoregulation, anti-viral and anti-coagulant. The current study was performed to investigate possible protective effects of fucoidan for sulfoxaflor-induced hematological/biochemical alterations and oxidative stress in the blood of male Swiss albino mice. For this purpose, sulfoxaflor was administered at a dose of 15 mg/kg/day (1/50 oral LD_50_), and fucoidan was administered at a dose of 50 mg/kg/day by oral gavage alone and combined for 24 h and 7 days. Hematological parameters (RBC, HGB, HCT, MCV, MCH, MCHC, Plt, WBC, Neu, Lym and Mon), serum biochemical parameters (AST, ALT, GGT, LDH, BUN, Cre and TBil), and serum oxidative stress/antioxidant markers (8-OHdG, MDA, POC and GSH) were analyzed. The results indicated that sulfoxaflor altered hematological and biochemical parameters and caused oxidative stress in mice; fucoidan ameliorated some hematological and biochemical parameters and exhibited a protective role as an antioxidant against sulfoxaflor-induced oxidative stress.

## 1. Introduction

In recent years, attention has been focused on whether naturally occurring compounds can modulate the effects of various toxic xenobiotics. These studies reported that antioxidant compounds obtained from natural sources prevented various tissues from toxic effects of xenobiotics [1]. One of these natural compounds is sulfated polysaccharide fucoidan isolated from brown macroalgae species such as *Macrocystis pyrifera*, *Fucus vesiculosus*, *Laminaria japonica*. Sulfated l-fucose is the main moiety of fucoidan with small proportions of glucose, galactose, xylose, mannose and uronic acids [2]. Fucoidan has several biological activities including antioxidant, antiproliferative, anti-inflammatory, antiangiogenic, antiviral, anticoagulant, antilipidemic, and immunomodulatory [3,4,5]. Thus, fucoidan has been the interest of many studies in pharmaceutical industries [6,7]. Fucoidan possesses strong in vitro and in vivo antioxidant activities [8,9]. Several studies have reported that fucoidan has a protective role against the toxicity of numerous xenobiotics including alcohol [10], acetaminophen [11], carbon tetrachloride [12], insecticide diazinon [13], fungicide cholorothalonil [14] due to its antioxidant, anti-inflammatory and anti-apoptotic properties.

Reactive oxygen species (ROS) that can damage cell function and structures via oxidation of DNA, lipids and proteins are formed during the biotransformation of xenobiotics. The cells have an antioxidant system for protection from ROS including catalase (CAT) and glutathione peroxidase (GPx), superoxide dismutase (SOD) and glutathione (GSH) [15]. Glutathione (GSH) as a low-molecular-mass thiol is the most important antioxidant which protects cells from the toxicity of xenobiotic electrophiles and oxidative damage. In addition, it maintains redox homeostasis in organisms via enzymatic or non-enzymatic reactions [16]. It was demonstrated that neonicotinoids may cause perturbation of the antioxidant system and they can induce oxidation of DNA, protein and lipids. The oxidation of these biomolecules by neonicotinoid-induced ROS can increase the levels of oxidative stress markers including 8-hydroxy-2′-deoxyguanosine (8-OHdG), malondialdehide (MDA) and protein carbonyl (POC) in cells [17]. Furthermore, numerous studies reported that neonicotinoids can affect hematological and biochemical parameters in mammals [18,19].

Neonicotinoid insecticides are currently sharing over 30% of the global market and are used abundantly worldwide as a veterinary medicine and for crop [20]. Several in vitro and in vivo studies indicated that neonicotinoids can have adverse effects on mammals [21]. Despite the well-defined mammalian toxicity of some neonicotinoids such as imidacloprid [22,23] and thiamethoxam [24,25] the toxic effects of the fourth generation of neonicotinoid–sulfoximine insecticides including sulfoxaflor have not been thoroughly investigated.

Sulfoxaflor (methyl(oxo){1-[6-(trifluoromethyl)-3-pyridyl]ethyl}-λ6-sulfanylidene]cyanamide) is a member of a newly developed neonicotinoid group of insecticides consisting of sulfoximine derivatives, and is mostly used to control a wide variety of insect species. Sulfoxaflor acts as a selective agonist for the nicotinic acetylcholine receptors (nAChRs) subtypes in insects and it exhibits different structure–activity relationships than neonicotinoids [26]. There are limited studies related to the acute and chronic toxic effects of sulfoxaflor on mammalian species. These studies reported that sulfoxaflor may also have carcinogenic [27,28] and teratogenic effects in mammals [29].

Previously, we demonstrated that sulfoxaflor led to oxidative stress and activation of GSH related antioxidants in the gill of zebrafish (*Danio rerio*) [30]. A mice study showed that fucoidan might play a modulatory role in oxidative stress and caspase-3 mRNA expression as an antioxidant in the brain of sulfoxaflor treated-mice [31]. As a continuation of our previous studies, the present study was aimed to investigate the possible protective effect of fucoidan against sulfoxaflor-induced hematological/biochemical alterations and oxidative stress in the blood of male Swiss albino mice.

## 2. Results

### 2.1. Changes in Hematological Parameters

The parameters were analyzed for determination of the effects of sulfoxaflor and fucoidan on hematological parameters in male mice including red blood cell (RBC) count, hemoglobin (HGB), hematocrit (HCT), mean corpuscular volume (MCV), mean corpuscular hemoglobin (MCH), mean corpuscular hemoglobin concentration (MCHC), platelets (Plt), white blood cell count (WBC), neutrophil (Neu %), lymphocyte (Lym %), monocyte (Mon %), eosinophil (Eos %).

The effects of sulfoxaflor and fucoidan on hematological parameters are presented in Table 1 and Table 2. Sulfoxaflor and fucoidan showed no significant effect on RBC count, HGB level, HCT, MCV, MCH, Neu count, Lym count, Mon count and Plt count after 24-h treatment, and on RBC, MCV, MCH, Neu, Lym, and Plt after 7-d treatment when compared with the control.

Sulfoxaflor treatment significantly decreased MCHC value and WBC count when compared with the control after 24-h and 7-d treatment periods (*p* < 0.05; Table 1 and Table 2). Furthermore, significant elevations in HGB were recorded for sulfoxaflor after the 7-d treatment period (*p* < 0.05; Table 2). On the other hand, fucoidan treatment normalized the WBC count in sulfoxaflor-treated mice after 24-h and 7-d treatment periods (Table 1 and Table 2). Significant elevations in HGB levels and HCT values were determined in sulfoxaflor + fucoidan-treated mice in the 7-d treatment period when compared with the control group (*p* < 0.05; Table 2). MCHC was significantly decreased in the sulfoxaflor + fucoidan-treated group when compared with the control in the 7-d treatment period (*p* < 0.05; Table 2). The Mon count (%) was decreased by sulfoxaflor + fucoidan treatment when compared with the control in the 7-d treatment period (*p* < 0.05; Table 2).

### 2.2. Changes in Serum Biochemical Markers of Liver and Kidney Functions

The following parameters were analyzed; serum aspartate aminotransferase (AST) activity, alanine aminotransferase (ALT) activity, γ-glutamyltransferase (GGT) activity, lactate dehydrogenase (LDH) activity, blood urea nitrogen (BUN) concentration, creatinine (Cre) concentration and total bilirubin (TBil) concentration for determination of the effects of sulfoxaflor and fucoidan on biochemical parameters. The effects of sulfoxaflor and fucoidan on biochemical parameters associated with liver and kidney function are demonstrated in Table 3. Sulfoxaflor had no significant effect on ALT and GGT activity after 24-h and 7-d treatment periods, but AST activity was significantly increased after the 24-h treatment period (*p* < 0.05; Table 3) when compared with the control. Similar to the observed changes in AST activity, sulfoxaflor caused significant elevations in LDH activity when compared with the control after the 24-h treatment period (*p* < 0.05; Table 3). Sulfoxaflor had no significant effect on Cre, BUN or TBil levels after 24-h or 7-d treatments. Fucoidan significantly decreased serum AST and LDH activities and BUN levels when compared with the control after the 7-d treatment period in the current study (*p* < 0.05; Table 3). Fucoidan treatment normalized sulfoxaflor-induced serum AST and LDH activities at the 24-h treatment period (*p* < 0.05; Table 3). Sulfoxaflor + fucoidan treatment also significantly decreased TBil levels when compared with the control during the 7-d treatment period (*p* < 0.05; Table 3).

### 2.3. Changes in Serum Oxidative Stress and Antioxidant Markers

**Figure 1:**
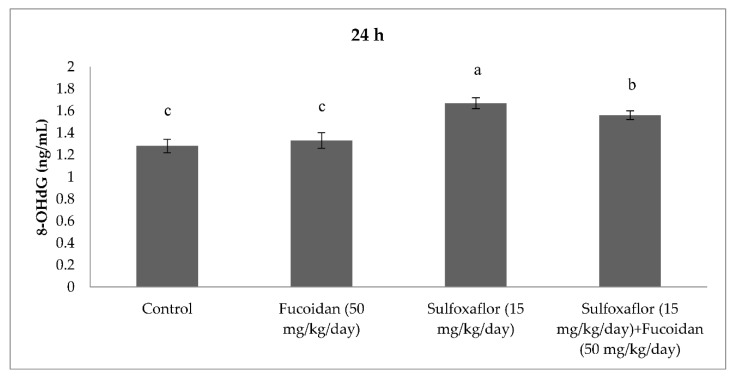
Effects of Sulfoxaflor and Fucoidan on 8-OHdG Levels (ng/mL) in Serum of Mice at 24-h Treatment Periods. Values are expressed as mean ± standard error. 8-OHdG: 8-hydroxy-2′-deoxyguanosine. Letters a, b and c show the differences between treatment groups and control. Data shown in different letters are significantly different at *p* < 0.05 level (*n* = 8). Duncan multiple comparison tests were used.

**Figure 2:**
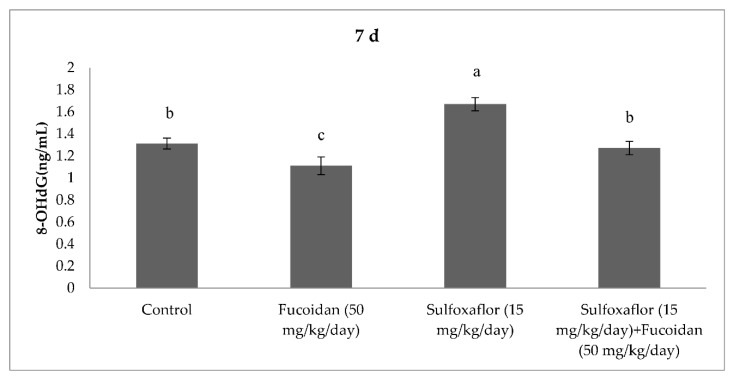
Effects of Sulfoxaflor and Fucoidan on 8-OHdG Levels (ng/mL) in Serum of Mice at 7-d Treatment Periods. Values are expressed as mean ± standard error. 8-OHdG: 8-hydroxy-2′-deoxyguanosine. Letters a, b and c show the differences between treatment groups and control. Data shown in different letters are significantly different at *p* < 0.05 level (*n* = 8). Duncan multiple comparison tests were used.

**Figure 3:**
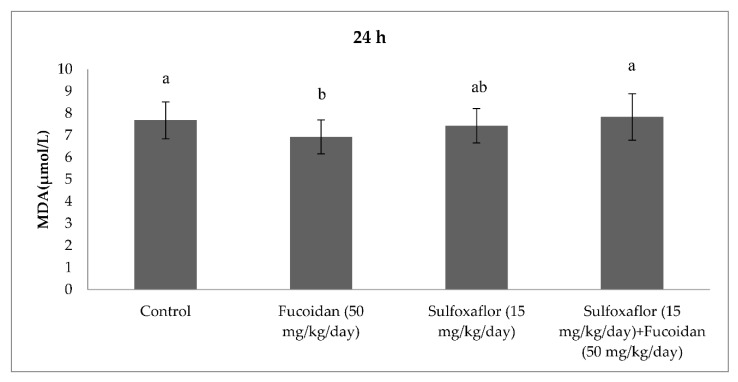
Effects of Sulfoxaflor and Fucoidan on MDA Levels (µmol/L) in Serum of Mice at 24-h Treatment Periods. Values are expressed as mean ± standard error. MDA: Malondialdehide. Letters a and b show the differences between treatment groups and control. Data shown in different letters are significantly different at *p* < 0.05 level (*n* = 8). Duncan multiple comparison tests were used.

**Figure 4:**
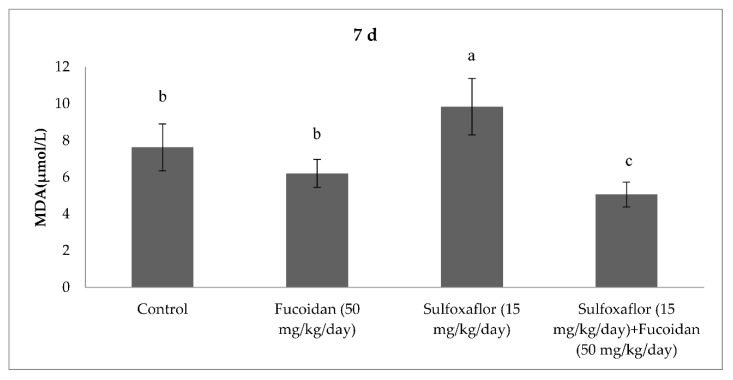
Effects of Sulfoxaflor and Fucoidan on MDA Levels (µmol/L) in Serum of Mice at 7-d Treatment Periods. Values are expressed as mean ± standard error. MDA: Malondialdehide. Letters a, b and c show the differences between treatment groups and control. Data shown in different letters are significantly different at *p* < 0.05 level (*n* = 8). Duncan multiple comparison tests were used.

**Figure 5:**
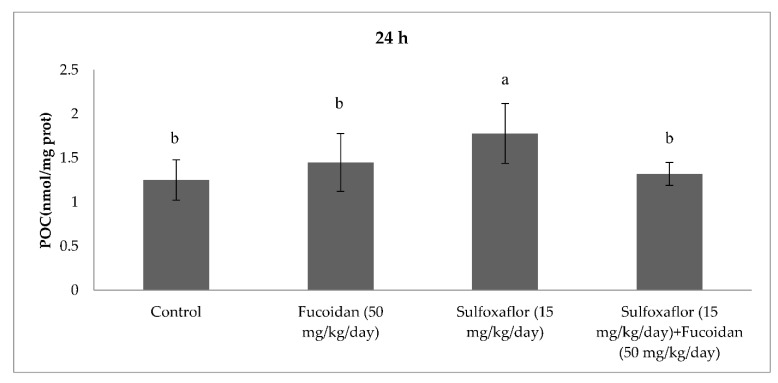
Effects of Sulfoxaflor and Fucoidan on POC Levels (nmol/mg protein) in Serum of Mice at 24-h Treatment Periods. Values are expressed as mean ± standard error. POC: Protein carbonyl. Letters a and b show the differences between treatment groups and control. Data shown in different letters are significantly different at *p* < 0.05 level (*n* = 8). Duncan multiple comparison tests were used.

**Figure 6:**
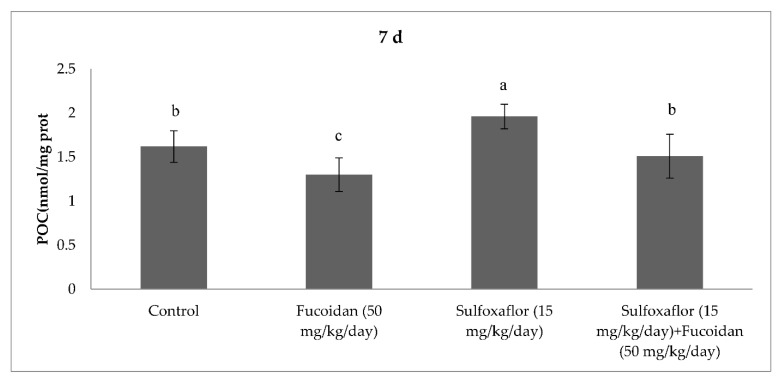
Effects of Sulfoxaflor and Fucoidan on POC Levels (nmol/mg protein) in Serum of Mice at 7-d Treatment Periods. Values are expressed as mean ± standard error. POC: Protein carbonyl. Letters a, b and c show the differences between treatment groups and control. Data shown in different letters are significantly different at *p* < 0.05 level (*n* = 8). Duncan multiple comparison tests were used.

**Figure 7:**
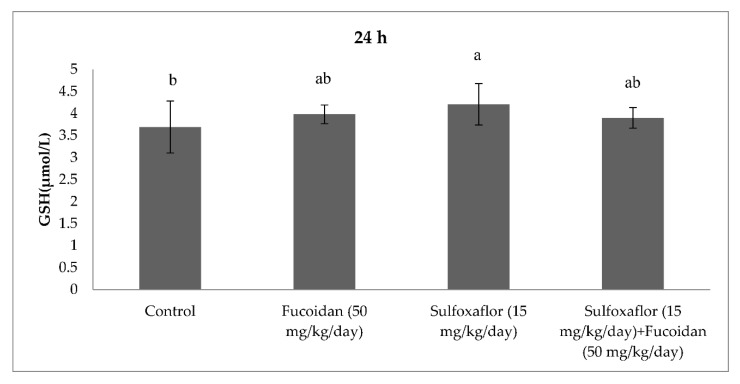
Effects of Sulfoxaflor and Fucoidan on GSH Levels (µmol/L)) in Serum of Mice at 24-h Treatment Periods. Values are expressed as mean ± standard error. Glutathione: GSH. Letters a and b show the differences between treatment groups and control. Data shown in different letters are significantly different at *p* < 0.05 level (*n* = 8). Duncan multiple comparison tests were used.

**Figure 8:**
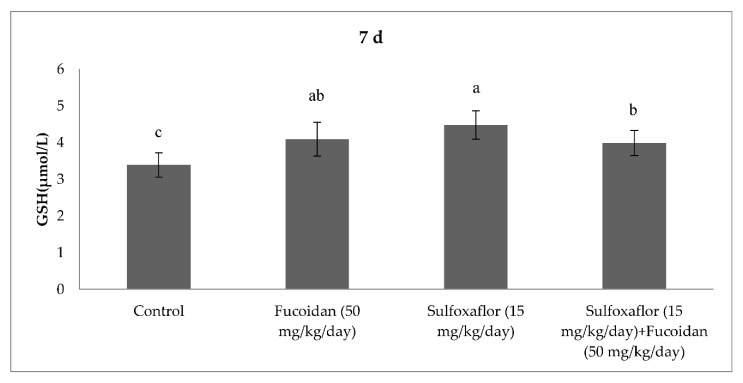
Effects of Sulfoxaflor and Fucoidan on GSH Levels (µmol/L) in Serum of Mice at 7-d Treatment Periods. Values are expressed as mean ± standard error. Glutathione: GSH. Letters a, b and c show the differences between treatment groups and control. Data shown in different letters are significantly different at *p* < 0.05 level (*n* = 8). Duncan multiple comparison tests were used.

The results obtained from serum oxidative and antioxidant parameters are presented in Figure 1, Figure 2, Figure 3, Figure 4, Figure 5, Figure 6, Figure 7 and Figure 8. Sulfoxaflor treatments led to significantly increased serum 8-OHdG levels when compared with the control after 24-h and 7-d treatment periods (*p* < 0.05; Figure 1 and Figure 2). Sulfoxaflor also significantly increased serum MDA levels when compared with the control at the 7-d treatment period (*p* < 0.05; Figure 4). Serum POC levels were significantly increased by sulfoxaflor when compared with the control after 24-h and 7-d treatments (*p* < 0.05; Figure 5 and Figure 6). Sulfoxaflor significantly increased serum GSH levels when compared with the control at 24-h and 7-d treatment periods (Figure 7 and Figure 8, *p* < 0.05). Fucoidan treatment significantly decreased serum 8-OHdG and POC levels when compared with the control after the 7-d treatment period (p < 0.05; Figure 2, Figure 3, Figure 4, Figure 5 and Figure 6). The MDA levels in serum were also decreased by fucoidan when compared with the control at 24-h (*p* < 0.05; Figure 3). Serum GSH levels were significantly increased by fucoidan after the 7-d treatment period when compared with the control (*p* < 0.05; Figure 8). Sulfoxaflor + fucoidan treatments significantly decreased serum 8-OHdG and MDA levels when compared with the sulfoxaflor treatment after 7-d (*p* < 0.05; Figure 2, Figure 3 and Figure 4). Similarly, sulfoxaflor + fucoidan treatments significantly decreased POC levels when compared with the sulfoxaflor treatment after the 24-h and 7-d treatment period (*p* < 0.05; Figure 5 and Figure 6). Sulfoxaflor + fucoidan also caused significant increases in GSH levels; however, the increase in GSH levels in the sulfoxaflor group was significantly higher than that in the sulfoxaflor + fucoidan treatment group (*p* < 0.05; Figure 7 and Figure 8).

## 3. Discussion

The results of this study revealed that sulfoxaflor altered hematological and biochemical parameters and caused oxidative stress in mice. Fucoidan had an ameliorative effect on some hematological and biochemical parameters and exhibited a protective role as an antioxidant against sulfoxaflor-induced oxidative stress.

The hematological parameters demonstrated that sulfoxaflor may cause toxic effects by inducing alterations in the MCHC, HGB and WBC in the mice. Similar to these findings, orally administered neonicotinoid insecticide imidacloprid caused significant elevations in the leukocyte count (WBC) and decreases in the erythrocyte count (RBC), HGB level, and erythrocyte sedimentation rate (ESR) in mice [32] whereas acetamiprid decreased total leukocyte count (TLC), HGB concentration, HCT value and total erythrocyte count (TEC), as well as caused variations in MCV, MCHC and MCH in mice [18]. Studies have reported that fucoidan is a very safe molecule that does not cause toxic effects in mammals at high doses [33,34,35]. Furthermore, fucoidan does not have toxic effects on hematological parameters at low doses in rats (50–150 mg/kg) [36]. Similar to this finding, fucoidan had no significant effect on hematological parameters at the dose of 50 mg/kg/day in male mice. In addition, fucoidan may provide moderate ameliorative effects on hematological parameters by increasing the WBC count and MCHC value. However, it did not have significant ameliorative effects on the HCT value, HGB level, Mon count (%), or Eos count (%) in sulfoxaflor-treated mice.

Serum ALT, AST and ALP activities and TBil levels have previously been reported to be increased in sulfoxaflor-treated mice; furthermore, treatment-related increases in the mean liver weights, hepatocellular hypertrophy in both sexes, and necrosis in male mice after 28 and 90 days of treatment have already been demonstrated [37,38]. Similar to these findings, sulfoxalor significantly increased serum AST activity at the 24-h treatment period in the present study. Bhardwaj et al. [39] and Zhang et al. [40] reported increases in AST activity in rats following the administration of imidacloprid and in mice following the administration of acetamiprid, respectively. Our results indicated that sulfoxaflor caused significant elevations in LDH activity similar to the observed changes in AST activity. Neonicotinoids have been shown to cause alterations in LDH activity in the tissues of the treated mammals and to induce hepatocellular damage [24,40]. Alterations in the biochemical markers analyzed in the current study emphasized that sulfoxaflor weakly affected biochemical markers associated with liver function in mice. Sulfoxaflor showed no significant effect on Cre, BUN or TBil levels. In contrast to these findings, some authors have determined that neonicotinoids cause elevations in BUN or Cre levels in serum or plasma in mammals [25,36,37]. The results of this study showed that sulfoxaflor did not have toxic effects on kidney function, as evidenced biochemically. Although fucoidan has not been reported to affect serum biochemical markers at low doses in mammals [12,41,42], it significantly decreased the serum AST and LDH activities and BUN levels in the current study. However, fucoidan treatment normalized sulfoxaflor-elevated serum AST and LDH activities. This finding indicated that fucoidan may play a protective role against sulfoxaflor-induced alterations in biochemical markers related to liver function. Rats treated with fucoidan exhibited significantly lower serum ALT, AST, ALP, and LDH activities than rats exposed to diazinon alone [13]. Aflatoxin B1 exposure caused dramatic increases in liver enzymes activities (AST, ALT, ALP and LDH), and fucoidan administration significantly decreased all the measured biochemical parameters [41].

The results showed that sulfoxaflor might cause ROS-induced oxidative DNA damage by increasing 8-OHdG levels in mice serum. Similar to these findings, previous studies have shown that neonicotinoids caused oxidative DNA damage by increasing 8-OHdG levels in fish and rats [43,44]. Sulfoxaflor also significantly increased serum MDA levels in the current study. We previously found that sulfoxaflor cause lipid peroxidation in the gills of zebrafish [30] and in the brain of male mice [31]. Consistent with this report, studies have demonstrated that neonicotinoids cause lipid peroxidation by increasing MDA or TBARS levels in different mammalian tissues [17]. In the present study, serum POC was also significantly increased by sulfoxaflor treatment. Although the oxidative stress effects of neonicotinoids have been extensively investigated, limited studies have considered the relationship between oxidative stress-induced effects of neonicotinoids and protein oxidation [45]. The results of the present study indicated that sulfoxaflor caused protein oxidation by increasing POC levels. Sulfoxaflor also significantly increased serum GSH levels. The increases in serum GSH levels might be related to scavenging of sulfoxaflor-induced ROS in mice. Thus, GSH-related antioxidants may play protective roles against the oxidative stress effects of sulfoxaflor.

The current study demonstrated that fucoidan treatment significantly decreased serum 8-OHdG, POC and MDA levels when compared with the control. Additionally, serum GSH levels were significantly increased by fucoidan when compared with the control. Alterations in serum oxidative stress and antioxidant markers demonstrated that fucoidan might play an antioxidant role by inhibiting oxidative stress markers and activating GSH in normal metabolic processes in male mice. Previous studies have reported that fucoidan supports the antioxidant system by activating GSH and enzymatic antioxidants such as GPx, CAT, and SOD in mammalian tissues, and it protects various tissues from oxidative damage caused by several toxicants in mice and rats [12,13,41,42]. Findings of this research showed that sulfoxaflor + fucoidan treatments significantly decreased 8-OHdG and MDA levels when compared with the sulfoxaflor treatment. Similarly, sulfoxaflor + fucoidan treatments significantly decreased POC levels when compared with the sulfoxaflor treatment. Consistent with the results of previous studies, the present study demonstrated that fucoidan provided effective protection as an antioxidant against the oxidative stress-producing effects of sulfoxaflor by supporting the GSH-related antioxidant system.

Significant relationships between structure and bioactivity of fucoidans have been reported previously [2]. Fucoidans are heterogeneous, and their source, processing techniques, molecular weight, sulphate content are among the factors that affect their structure and activity [46,47,48]. Although the molecular weight of fucoidans varies from 43 up to 1600 kDa [49], the bioactivity of the low-molecular-weight fucoidans have been focused in the most of the studies. The variation among fucoidans is displayed in fucose (25–93%), sulfate content (9–40%), uronic acid (up to 25%), and neutral sugars [50]. Most fucoidans isolated from macrolage species exhibit complex chemical compositions. Other monosaccharides including mannose, galactose, glucose, xylose, and uronic acids, even acetyl groups and protein could be contained by fucoidans [2]. Fucoidans have been reported to have excellent antioxidant properties and have a great potential for preventing free radical-mediated diseases. Studies indicated that molecular weight and sulfate content of fucoidan were related to its antioxidant activity [51,52,53]. Fucoidan obtained from *F. vesiculosus* was composed of 44.1% fucose, 26.3% sulfate, 31.1% ash and aminoglucose [2]. Micheline et al. [54] reported that the formation of hydroxyl radical and superoxide radical was inhibited by fucoidan obtained from *F. vesiculosus*. Recent reports showed that fucoidan isolated from *F. vesiculosus* had a high molecular weight with an average of 735 kDa [55] and had a good radical scavenging activity [5]. Consistent with these previous reports, the findings of the present study suggested that fucoidan isolated from *F. vesiculosus* may have a strong antioxidant activity in vivo.

Several studies conducted in mammals revealed that fucoidan had various biological activities in tissues and organs [4,47,56,57]. A limited number of studies for the pharmacokinetics and tissue distribution of fucoidan have been reported following an oral administration in mammals [5]. This report indicated that fucoidan extracted from *F. vesiculosus* accumulated in the kidneys, spleen and liver and showed a relatively long absorption time and extended circulation in blood after a single-dose oral administration in rats. In additional, fucoidan uptake and urinary excretion have been demonstrated in humans after oral administration [58,59]. Mentioned human and rodent studies indicated that the source of fucoidan and its molecular weight as well as metabolism rate in organisms may affect pharmacokinetics and tissue distribution of fucoidan. In the current study, the protective effects of fucoidan against sulfoxaflor toxicity may support the use of oral fucoidan in mammals.

## 4. Materials and Methods

### 4.1. Animals

Sixty-four healthy male mice (Swiss albino) weighing 26 ± 2 g and 8–10 months of age were procured from the Faculty of Medicine Experimental Medicine Research and Application Centre, Cukurova University (Turkey). The animals were acclimatized to the laboratory conditions for at least one week prior to experiments. The temperature and relative humidity of the animal room were maintained at 22 ± 2 °C and 50% to 60%, respectively. The photoperiod of light and dark cycle of 12 h each was applied. The control and test animals were given food in pellet form (TAVAŞ^®^) and water ad libitum. All test procedures were reviewed and approved by the Ethics Committee of the Cukurova University Faculty of Medicine Experimental Medicine Research and Application Centre, Turkey (approval date: 4 November 2019, approval code: 6).

### 4.2. Chemicals, Reagents, Kits

Commercial formulation of sulfoxaflor called Transform 500WG (%50 *w*/*w* active ingredient), ([methyl (oxo) {1-[6-(trifluoromethyl)-3-pyridyl] ethyl}-λ6-sulfanylidene] cyanamide, CAS number: 946578-00-3) was obtained from a distributor company in Turkey. Fucoidan (*F. vesiculosus*, Sigma F5631, Sigma-Aldrich Co., St. Louis, MO, USA) was purchased. This commercially available fucoidan consisted of 138.7 mg/g fucose, 341.6 mg/g sulfate, 27.9 mg/g galactose, 18.5 mg/g glucronic acide, 12.8 mg/g xylose, 2.8 mg/g arabinose, 2.5 mg/g glucose, 2 mg/g rhamnose, 0.2 mg/g mannose [60] and, its molecular weight was between 105–117 kDa [61].

Hematology reagents were purchased from Mindray Bio-Medical Electronics Co., Ltd. (Shenzhen, China). Slides used for determination of biochemical parameters in serum were obtained from Fujifilm Holding Corp (Tokyo, Japan). Kits used for analyzing oxidative stress markers in serum were supplied from Elabscience, Inc. (Wuhan, Hubei, China). All other chemicals were purchased from Sigma-Aldrich Co. (St. Louis, MO, USA), and Merck&Co.Inc (Merck, Darmstadt, Germany).

### 4.3. Experimental Design

Acute oral toxicity tests (24-h, 7-d) were conducted according to the OECD protocols [62]. Sixty-four mice were randomly divided into eight experimental groups (*n* = 8). Experimental Groups included: (1) Control Group: Physiological saline (24-h), (2) Fucoidan Group: 50 mg/kg/day (24-h), (3) Sulfoxaflor Group: 15 mg/kg/day (24-h), (4) Sulfoxaflor + fucoidan Group: 15 mg/kg/day + 50 mg/kg/day (24-h), (5) Control Group: Physiological saline (7-d), (6) Fucoidan Group: 50 mg/kg/day (7-d), (7) Sulfoxaflor Group: 15 mg/kg/day (7-d), (8) Sulfoxaflor + fucoidan Group: 15 mg/kg/day + 50 mg/kg/day (7-d). Sulfoxaflor and fucoidan were dissolved in physiological saline. Sulfoxaflor was administered to mice at a dose of 15 mg/kg/day (1/50 oral LD_50_) by oral gavage [63]. The dose of sulfoxaflor used in the current toxicity tests was determined by evaluating the clinical toxicity symptoms of mice during the 7-day treatment period in a preliminary study [31]. Fucoidan was applied at a dose of 50 mg/kg/day by oral gavage by evaluating its antioxidant activity based on previous studies conducted with mice [64,65]. Fucoidan was applied 2 h before sulfoxaflor administration.

Clinical observation of mice after each treatment (survival, body mass, clinical conditions) was recorded periodically (2-h). No deaths and no clinical signs of toxic effects were observed for the administered dose of the sulfoxafor (15 mg/kg/day). Mice were removed from cages at the end of each treatment period, anaesthetized with ketamine/xylazine. Blood samples were collected by cardiac puncture. Blood portion was collected upon anticoagulant (EDTA) for analyzing the hematological parameters. Serum samples were obtained by centrifugation of each blood sample at 1500× *g* for 5 min using a centrifuge (Hettich Universal 320R, Hettichlab, Germany). Non-hemolyzed serum was stored at −80 °C until analysis. At the end of each treatment period, the hematological parameters (RBC, HGB, HCT, MCV, MCH, MCHC, Plt, WBC, Neu, Lym and Mon), serum biochemical markers (AST, ALT, GGT, LDH, BUN, Cre and TBil) and, serum oxidative stress markers (8-OHdG, MDA and POC levels) and antioxidant marker (GSH levels) were analyzed.

### 4.4. Determination of Hematological Parameters

Hematological parameters (RBC, HGB, HCT, MCV, MCH, MCHC, Plt, WBC, Neu, Lym and Mon) were analyzed using Mindray hematology reagents and the Mindray Vet-5300 Auto Hematology Analyzer according to the providers’ instructions.

### 4.5. Determination of Biochemical Parameters in Serum

Biochemical parameters (AST, ALT, GGT, LDH, BUN, Cre and TBil) analyzed using Fujifilm Dri-Chem Slides and the Fuji Dri-Chem 4000i Automated Clinical Chemistry Analyzer according to the providers’ instructions.

### 4.6. Determination of Oxidative Stress and Antioxidant Markers

The levels of 8-OHdG in serum samples were determined according to the protocol specified in the ELISA kit (Elabscience, E-EL-0028) using Microplate Reader (Biotek ELx800, Biotek Intruments, Inc., Winooski, VT, USA). MDA and GSH levels in serum samples were analyzed according to the protocol specified in the colorimetric assay kit (Elabscience, E-BC-K025-M and E-BC-K030-M, respectively) using a Microplate Reader (Biotek ELx800, Biotek Intruments, Inc., Winooski, VT, USA). The levels of POC in serum samples were measured according to the protocol specified in the colorimetric assay kit (Elabscience, E-BC-K117-S) using a UV-Visible spectrophotometer (Shimadzu UV-1700, Shimadzu, Kyoto, Japan).

### 4.7. Determination of Protein Levels

Total protein levels in serum samples were determined with BCA method using a colorimetric assay kit (Elabscience, E-BC-K318-M) and Microplate Reader. Total protein levels were used for calculation of POC levels in serum samples.

### 4.8. Statistical Analyzes

Data were statistically analyzed with analysis of variance (ANOVA) using SPSS software version 22.0 (IBM SPSS Statistics, NY, USA). Duncan multiple comparison tests were conducted for mean separation of control and treated groups at significance level *p*< 0.05. The results were expressed as mean ± standard error (SE).

## 5. Conclusions

Fucoidans are the sulphated polysaccharides mostly isolated from brown macroalgea species and possess various therapeutic activities which make them important for biomedical applications. The results of the current study showed that sulfoxaflor caused alterations in hematological parameters and biochemical markers of liver function in male mice. Sulfoxaflor also caused oxidative stress by inducing DNA, protein, and lipid oxidation. In the light of the aforementioned results, oral fucoidan administration might exhibit a protective role against the sulfoxaflor-induced hematological/biochemical alterations and oxidative toxicity of sulfoxaflor.

## Figures and Tables

**Table 1:** Effects of sulfoxaflor and fucoidan on hematological parameters at 24-h treatment period in mice.

24-h	Control	Fucoidan (50 mg/kg/day)	Sulfoxaflor (15 mg/kg/day)	Sulfoxaflor (15 mg/kg/day) + Fucoidan (50 mg/kg/day)
RBC (10^12^/L)	7.01 ± 1.01 ^a^	6.69 ± 0.49 ^a^	7.43 ± 0.73 ^a^	7.31 ± 0.81 ^a^
HGB (g/dL)	10.56 ± 1.49 ^ab^	10.51 ± 0.27 ^b^	11.32 ± 0.81 ^a^	10.98 ± 0.06 ^a^
HCT	0.35 ± 0.05 ^a^	0.34 ± 0.02 ^a^	0.38 ± 0.03 ^a^	0.36 ± 0.04 ^a^
MCV (fL)	49.98 ± 2.16 ^a^	50.31 ± 3.25 ^a^	52.15 ± 4.6 ^a^	50.56 ± 2.17 ^a^
MCH (pg)	15.06 ± 0.74 ^a^	15.11 ± 0.8 ^a^	15.28 ± 1.16 ^a^	15.05 ± 0.77 ^a^
MCHC (g/dL)	30.17 ± 0.7 ^a^	30.09 ± 0.74 ^a^	29 ± 0.09 ^b^	29.73 ± 0.69 ^ab^
Plt (10^9^/L)	633.62 ± 142.71 ^ab^	473.93 ± 204.97 ^b^	677.31 ± 197.2 ^a^	675.93 ± 140.58 ^a^
WBC (10^9^/L)	6.49 ± 0.26 ^a^	6.07 ± 0.33 ^ab^	5.41 ± 1.11 ^b^	6.46 ± 0.65 ^a^
Neu (%)	0.393. ± 0.10 ^a^	0.427 ± 0.16 ^a^	0.306 ± 0.08 ^a^	0.364 ± 0.11 ^a^
Lym (%)	0.538 ± 0.12 ^a^	0.499 ± 0.18 ^a^	0.618 ± 0.11 ^a^	0.891 ± 0.08 ^a^
Mon (%)	0.048 ± 0.016 ^a^	0.037 ± 0.15 ^a^	0.052 ± 0.035 ^a^	0.034 ± 0.02 ^a^
Eos (%)	0.0187 ± 0.012 ^a^	0.0194 ± 0.039 ^a^	0.0215 ± 0.011 ^b^	0.0277 ± 0.008 ^b^

RBC: red blood cell count, HGB: hemoglobin, HCT: hematocrit, MCV: mean corpuscular volume, MCH: mean corpuscular hemoglobin, MCHC: mean corpuscular hemoglobin concentration, Plt: platelets, WBC: white blood cell count, Neu %: neutrophil, Lym %: lymphocyte, Mon %: monocyte, Eos %: eosinophil. Values are expressed as mean ± standard error. Letters ^a^ and ^b^ show the differences between treatment groups at the same treatment period. Data shown in different letters are significantly different at *p* < 0.05 level (*n* = 8). Duncan multiple comparison tests were used.

**Table 2:** Effects of sulfoxaflor and fucoidan on hematological parameters at 7-d treatment period in mice.

7-d	Control	Fucoidan (50 mg/kg/day)	Sulfoxaflor (15 mg/kg/day)	Sulfoxaflor (15 mg/kg/day) + Fucoidan (50 mg/kg/day)
RBC (10^12^/L)	7.41 ± 0.048 ^a^	7.31 ± 0.32 ^a^	7.65 ± 0.29 ^a^	9.14 ± 0.02 ^a^
HGB (g/dL)	11.68 ± 0.7 ^c^	11.0 ± 0.38 ^ab^	12.12 ± 0.5 ^b^	14.68 ± 1.35 ^a^
HCT	0.38 ± 0.02 ^b^	0.37 ± 0.03 ^ab^	0.41 ± 0.02 ^b^	0.50 ± 0.04 ^a^
MCV (fL)	51.91 ± 0.64 ^ab^	46.92 ± 4.64 ^a^	51.21 ± 0.68 ^b^	54.77 ± 2.01 ^a^
MCH (pg)	15.68 ± 0.24 ^a^	16.95 ± 1.18 ^a^	15.83 ± 0.28 ^a^	15.93 ± 0.52 ^a^
MCHC (g/dL)	30.25 ± 0.38 ^a^	30.23 ± 0.23 ^a^	29.22 ± 0.21 ^b^	29.15 ± 0.15 ^b^
Plt (10^9^/L)	583.93 ± 200.97 ^a^	522.31 ± 136.94 ^a^	626.62 ± 142.67 ^a^	628.25 ± 146.06 ^a^
WBC (10^9^/L)	6.50 ± 0.3 ^a^	6.37 ± 0.3 ^a^	4.38 ± 0.26 ^b^	5.88 ± 0.35 ^a^
Neu (%)	0.438 ± 0.115 ^a^	0.389 ± 0.141 ^a^	0.303 ± 0.132 ^a^	0.411 ± 0.134 ^a^
Lym (%)	0.489 ± 0.135 ^a^	0.550 ± 0.165 ^a^	0.605 ± 0.128 ^a^	0.467 ± 0.124 ^a^
Mon (%)	0.053 ± 0.031 ^b^	0.044 ± 0.023 ^b^	0.076 ± 0.047 ^ab^	0.105 ± 0.063 ^a^
Eos (%)	0.0183 ± 0.005 ^ab^	0.0176 ± 0.005 ^ab^	0.0131 ± 0.006 ^b^	0.0242 ± 0.012 ^a^

RBC: red blood cell count, HGB: hemoglobin, HCT: hematocrit, MCV: mean corpuscular volume, MCH: mean corpuscular hemoglobin, MCHC: mean corpuscular hemoglobin concentration, Plt: platelets, WBC: white blood cell count, Neu %: neutrophil, Lym %: lymphocyte, Mon %: monocyte, Eos %: eosinophil. Values are expressed as mean ± standard error. Letters ^a^, ^b^ and ^c^ show the differences between treatment groups at the same treatment period. Data shown in different letters are significantly different at *p* < 0.05 level (*n* = 8). Duncan multiple comparison tests were used.

**Table 3:** Effects of sulfoxaflor and fucoidan on biochemical parameters in serum of mice at 24-h and 7-d treatment periods.

24-h	Control	Fucoidan (50 mg/kg/day)	Sulfoxaflor (15 mg/kg/day)	Sulfoxaflor (15 mg/kg/day) + Fucoidan (50 mg/kg/day)
AST (U/L)	50.25 ± 6.71 ^b^	53 ± 5.12 ^b^	66.25 ± 8.03 ^a^	55.75 ± 6.36 ^b^
ALT) (U/L)	69 ± 11.1 ^a^	68.75 ± 6.18 ^a^	74.87 ± 12.59 ^a^	68.25 ± 9.03 ^a^
GGT (U/L)	13.06 ± 0.80 ^a^	13.68 ± 0.54 ^a^	14.87 ± 12.59 ^a^	13.55 ± 9.03 ^a^
LDH (U/L)	516.12 ± 115.83 ^b^	532.25 ± 142.59 ^b^	1171.12 ± 361.65 ^a^	439.87 ± 149.82 ^b^
Cre (mg/dL)	0.49 ± 0.047 ^a^	0.51 ± 0.09 ^a^	0.53 ± 0.063 ^a^	0.50 ± 0.03 ^a^
BUN (mg/dL)	57.5 ± 7.75 ^a^	56.25 ± 5.99 ^a^	57.12 ± 12.4 ^a^	53.12 ± 3.97 ^a^
TBil (mg/dL)	0.78 ± 0.31 ^a^	0.94 ± 0.72 ^a^	0.71 ± 0.15 ^a^	0.71 ± 0.62 ^a^
**7-d**				
AST (U/L)	53.25 ± 8.13 ^a^	37.87 ± 4.51 ^b^	40.01 ± 4.79 ^ab^	48.5 ± 7.38 ^a^
ALT (U/L)	73.5 ± 12.31 ^a^	62.25 ± 4.46 ^a^	68.25 ± 23.33 ^a^	76.5 ± 11.45 ^a^
GGT (U/L)	15 ± 2.65 ^a^	14.75 ± 2.19 ^a^	14.92 ± 2.29 ^a^	15.03 ± 1.77 ^a^
LDH (U/L)	442.5 ± 162.43 ^a^	204.75 ± 38.27 ^b^	322.12 ± 234.38 ^ab^	492 ± 276.16 ^a^
Cre (mg/dL)	0.49 ± 0.05 ^a^	0.47 ± 0.04 ^a^	0.50 ± 0.04 ^a^	0.49 ± 0.03 ^a^
BUN (mg/dL)	60.08 ± 5.59 ^a^	51 ± 1.51 ^b^	57.2 ± 11.04 ^ab^	55.6 ± 6.06 ^ab^
TBil (mg/dL)	0.9 ± 0.27 ^a^	0.67 ± 0.12 ^b^	0.77 ± 0.19 ^ab^	0.66 ± 0.11 ^b^

AST: aspartate aminotransferase activity, ALT: alanine aminotransferase activity, GGT: γ-glutamyltransferase activity, LDH: lactate dehydrogenase activity, BUN: blood urea nitrogen concentration, Cre: creatinine concentration, TBil: total bilirubin concentration. Values are expressed as mean ± standard error. Letters ^a^ and ^b^ show the differences between treatment groups at the same treatment period. Data shown in different letters are significantly different at *p* < 0.05 level (*n* = 8). Duncan multiple comparison tests were used.

## Data Availability

The data has been presented in main text.

## References

[1] Zymanczyk–Duda E., Szmigiel–Merena B., Brzezinska–Rodak M. (2018). Natural antioxidants–properties and possible applications. J. Appl. Biotechnol. Bioeng..

[2] Li B., Lu F., Wei X., Zhao R. (2008). Fucoidan: Structure and bioactivity. Molecules.

[3] Krylova N.V., Ermakova S.P., Lavrov V.F., Leneva I.A., Kompanets G.G., Iunikhina O.V., Nosik M.N., Ebralidze L.K., Falynskova I.N., Silchenko A.S. (2020). The comparative analysis of antiviral activity of native and modified fucoidans from brown algae *Fucus evanescens* in vitro and in vivo. Mar. Drugs.

[4] Luthuli S., Wu S., Cheng Y., Zheng X., Wu M., Tong H. (2019). Therapeutic effects of fucoidan: A review on recent studies. Mar. Drugs.

[5] Pozharitskaya O.N., Obluchinskaya E.D., Shikov A.N. (2020). Mechanisms of bioactivities of fucoidan from the brown seaweed *Fucus vesiculosus* L. of the Barents sea. Mar. Drugs.

[6] Kwak J.Y. (2014). Fucoidan as a marine anticancer agent in preclinical development. Mar. Drugs.

[7] Zorofchian Moghadamtousi S., Karimian H., Khanabdali R., Razavi M., Firoozinia M., Zandi K., Abdul Kadir H. (2014). Anticancer and antitumor potential of fucoidan and fucoxanthin, two main metabolites isolated from brown algae. Sci. World J..

[8] Luo D., Zhang Q., Wang H., Cui Y., Sun Z., Yang J., Zheng Y., Jia J., Yu F., Wang X. (2009). Fucoidan protects against dopaminergic neuron death in vivo and in vitro. Eur. J. Pharmacol..

[9] Omar H.E.D.M., Eldien H.M.S., Badary M.S., Al–Khatib B.Y., Abd Elgaffar S.K. (2013). The immunomodulating and antioxidant activity of fucoidan on the splenic tissue of rats treated with cyclosporine A. J. Basic Appl. Zool..

[10] Lim J.D., Lee S.R., Kim T., Jang S.A., Kang S.C., Koo H.J., Sohn E., Bak J.P., Namkoong S., Kim H.K. (2015). Fucoidan from *Fucus vesiculosus* protects against alcohol–induced liver damage by modulating inflammatory mediators in mice and HepG2 cells. Mar. Drugs.

[11] Hong S.W., Lee H.S., Jung K.H., Lee H., Hong S.S. (2012). Protective effect of fucoidan against acetaminophen–induced liver injury. Arch. Pharm. Res..

[12] AlKahtane A.A., Abushouk A.I., Mohammed E.T., ALNasser M., Alarifi S., Ali D., Alessia M.S., Almeer R.S., AlBasher G., Alkahtani S. (2020). Fucoidan alleviates microcystin-LR-induced hepatic, renal, and cardiac oxidative stress and inflammatory injuries in mice. Environ. Sci. Pollut. Res. Int..

[13] Abdel-Daim M.M., Abushouk A.I., Bahbah E.I., Bungău S.G., Alyousif M.S., Aleya L., Alkahtani S. (2020). Fucoidan protects against subacute diazinon-induced oxidative damage in cardiac, hepatic, and renal tissues. Environ. Sci. Pollut. Res. Int..

[14] Mahgoub H.A., El–Adl M.A.M., Martyniuk C.J. (2021). Fucoidan ameliorates acute and sub–chronic *in vivo* toxicity of the fungicide cholorothalonil in *Oreochromis niloticus* (Nile tilapia). Comp. Biochem. Phys. C.

[15] Pisoschi A.M., Pop A., Iordache F., Stanca L., Predoi G., Serban A.I. (2021). Oxidative stress mitigation by antioxidants—An overview on their chemistry and influences on health status. Eur. J. Med. Chem..

[16] Bjørklund G., Peana M., Maes M., Dadar M., Severin B. (2021). The glutathione system in Parkinson’s disease and its progression. Neurosci. Biobehav. Rev..

[17] Wang X., Anadón A., Wu Q., Qiao F., Ares I., Martínez-Larrañaga M.R., Yuan Z., Martínez M.A. (2018). Mechanism of neonicotinoid toxicity: Impact on Oxidative stress and metabolism. Annu. Rev. Pharmacol. Toxicol..

[18] Singh T.B., Mukhopadhayay S.K., Sar T.K., Ganguly S. (2012). Acetamiprid induces toxicity in mice under experimental conditions with prominent effect on the hematobiochemical parameters. J. Drug Metab. Toxicol..

[19] Chakroun S., Ezzi L., Grissa I., Kerkeni E., Neffati F., Bhouri R., Sallem A., Najjar M.F., Hassine M., Mehdi M. (2016). Hematological, biochemical, and toxicopathic effects of subchronic acetamiprid toxicity in Wistar rats. Environ. Sci. Pollut. R.

[20] Jeschke P., Nauen R., Schindler M., Alfred M., Elbert A. (2011). Overview of the status and global strategy for neonicotinoids. J. Agric. Food Chem..

[21] Gibbons D., Morrissey C., Mineau P. (2015). A review of the direct and indirect effects of neonicotinoids and fipronil on vertebrate wildlife. Environ. Sci. Pollut. R.

[22] El–Gendy K.S., Aly N.M., Mahmoud F.H., Kenawy A., El–Sebae A.K.H. (2010). The role of vitamin C as antioxidant in protection of oxidative stress induced by imidacloprid. Food Chem. Toxicol..

[23] Kapoor U., Srivastava M., Srivastava L. (2011). Toxicological impact of technical imidacloprid on ovarian morphology, hormones and antioxidant enzymes in female rats. Food Chem. Toxicol..

[24] Khaldoun–Oularbi H., Bouzid N., Boukreta S., Makhlouf C., Derriche F., Djennas N. (2017). Thiamethoxam Actara^®^ induced alterations in kidney liver cerebellum and hippocampus of male rats. J. Xenobiot..

[25] Rodrigues K.J., Santana M.B., Do Nascimento J.L., Picanço–Diniz D.L., Maués L.A., Santos S.N., Ferreira V.M., Alfonso M., Durán R., Faro L.R. (2010). Behavioral and biochemical effects of neonicotinoid thiamethoxam on the cholinergic system in rats. Ecotoxicol. Environ. Saf..

[26] Zhu Y., Loso M.R., Watson G.B., Sparks T.C., Rogers R.B., Huang J.X., Gerwick B.C., Babcock J.M., Kelley D., Hegde V.B. (2011). Discovery and characterization of sulfoxaflor, a novel insecticide targeting sap-feeding pests. J. Agric. Food Chem..

[27] LeBaron M.J., Geter D.R., Rasoulpour R.J., Gollapudi B.B., Thomas J., Murray J., Kan H.L., Wood A.J., Elcombe C., Vardy A. (2013). An integrated approach for prospectively investigating a mode-of-action for rodent liver effects. Toxicol. Appl. Pharmacol..

[28] Rasoulpour R.J., Terry C., LeBaron M.J., Stebbins K., Ellis-Hutchings R.G., Billington R. (2014). Mode-of-action and human relevance framework analysis for rat Leydig cell tumors associated with sulfoxaflor. Crit. Rev. Toxicol..

[29] Rasoulpour R.J., Ellis-Hutchings R.G., Terry C., Millar N.S., Zablotny C.L., Gibb A.V., Marshall T., Collins E.W., Carney E.W., Billington R. (2012). A novel mode-of-action mediated by the fetal muscle nicotinic acetylcholine receptor resulting in developmental toxicity in rats. Toxicol. Sci..

[30] Piner Benli P., Celik M. (2021). Glutathione and its dependent enzymes’ modulatory responses to neonicotinoid insecticide sulfoxaflor-induced oxidative damage in zebrafish in vivo. Sci. Prog..

[31] Piner Benli P., Kaya M., Coskun C. (2021). Fucoidan modulated oxidative stress and caspase-3 mRNA expression induced by sulfoxaflor in the brain of mice. Neurotox. Res..

[32] Kataria S.K., Chhillar A.K., Kumar A., Tomar M., Malik V. (2016). Cytogenetic and hematological alterations induced by acute oral exposure of imidacloprid in female mice. Drug Chem. Toxicol..

[33] Li N., Zhang Q., Song J. (2005). Toxicological evaluation of fucoidan extracted from Laminaria japonica in Wistar rats. Food Chem. Toxicol..

[34] Phull A.R., Majid M., Haq I.U., Khan M.R., Kim S.J. (2017). In vitro and in vivo evaluation of anti-arthritic, antioxidant efficacy of fucoidan from *Undaria pinnatifida* (Harvey) Suringar. Int. J. Biol. Macromol..

[35] Ramu S., Murali A., Narasimhaiah G., Jayaraman A. (2020). Toxicological evaluation of *Sargassum wightii* greville derived fucoidan in wistar rats: Hematological, biochemical and histopathological evidences. Toxicol. Rep..

[36] Chung H.J., Jeun J., Houng S.J., Jun H.J., Kweon D.K., Lee S.J. (2010). Toxicological evaluation of fucoidan from *Undaria pinnatifidain* vitro and in vivo. Phytother. Res..

[37] Thomas J., Murray J.A., Saghir S.A., Yano B.L. (2010). Xr-208: 90-Day Dietary Toxicity Study in CD-1 Mice.

[38] Thomas J., Dryzga M.D., Saghir S.A., McClymont E.L., Quast J.F. (2008). Xr-208: 4-Week Repeated Dose Dietary Toxicity Study in crl: CD1(icr) Mice.

[39] Bhardwaj S., Srivastava M.K., Kapoor U., Srivastava L.P. (2010). A 90 days oral toxicity of imidacloprid in female rats: Morphological, biochemical and histopathological evaluations. Food Chem. Toxicol..

[40] Zhang J.J., Wang Y., Xiang H.Y., Li M.X., Li W.H., Ma K.G., Wang X.Z., Zhang J.H. (2011). Oxidative stress: Role in acetamiprid-induced impairment of the male mice reproductive system. Agric. Sci. China..

[41] Abdel-Daim M.M., Abdeen A., Jalouli M., Abdelkader A., Megahed A., Alkahtane A., Almeer R., Alhoshani N.M., Al-Johani N.S., Alkahtani S. (2021). Fucoidan supplementation modulates hepato-renal oxidative stress and DNA damage induced by aflatoxin B1 intoxication in rats. Sci. Total Environ..

[42] Aleissa M.S., Alkahtani S., Abd Eldaim M.A., Ahmed A.M., Bungău S.G., Almutairi B., Bin-Jumah M., AlKahtane A.A., Alyousif M.S., Abdel-Daim M.M. (2020). Fucoidan ameliorates oxidative stress, inflammation, DNA Damage, and hepatorenal injuries in diabetic rats intoxicated with aflatoxin B_1_. Oxidative Med. Cell. Longev..

[43] Topal A., Alak G., Ozkaraca M., Yeltekin A.C., Comaklı S., Acıl G., Kokturk M., Atamanalp M. (2017). Neurotoxic responses in brain tissues of rainbow trout exposed to imidacloprid pesticide: Assessment of 8–hydroxy–2–deoxyguanosine activity, oxidative stress and acetylcholinesterase activity. Chemosphere.

[44] Mohamed A.A.R., Mohamed W.A.M., Khater S.I. (2017). Imidacloprid induces various toxicological effects related to the expression of 3 beta-hsd, nr5a1, and ogg1 genes in mature and immature rats. Environ. Pollut..

[45] Mishchuk O.V., Stoliar O.B. (2008). The effect of pesticide acetamiprid on biochemical markers in tissues of fresh water bivalve mussels anodonta cygnea l. (unionidae). Ukr. Biochem. J..

[46] Wang Y., Xing M., Cao Q., Ji A., Liang H., Song S. (2019). Biological activities of fucoidan and the factors mediating its therapeutic effects: A review of recent studies. Mar. Drugs.

[47] Fitton J.H., Stringer D.N., Park A.Y., Karpiniec S.S. (2019). Therapies from fucoidan: New developments. Mar. Drugs.

[48] Jönsson M., Allahgholi L., Sardari R.R., Hreggviðsson G.O., Nordberg Karlsson E. (2020). Extraction and modification of macroalgal polysaccharides for current and next-generation applications. Molecules.

[49] Fletcher H.R., Biller P., Ross A.B., Adams J.M.M. (2017). The seasonal variation of fucoidan within three species of brown macroalgae. Algal Res..

[50] Usov A.I., Bilan M.I. (2009). Fucoidans—Sulfated polysaccharides of brown algae. Russ. Chem. Rev..

[51] Zhao X., Xue C.H., Cai Y.P., Wang D.F., Fang Y. (2005). The study of antioxidant activities of fucoidan from Laminaria japonica. High Technol. Lett..

[52] Li L.H., Xue C.H., Xue Y., Li Z.J., Fu X.Y. (2006). The effects of fucoidans from *Laminaria japonica* on AAPH mediated oxidation of human low-density lipoprotein. Acta Oceanol. Sin..

[53] Wang J., Zhang Q., Zhang Z., Li Z. (2008). Antioxidant activity of sulfated polysaccharide fractions extracted from *Laminaria japonica*. Int. J. Biol. Macromol..

[54] Micheline R.S., Cybelle M., Celina G.D., Fernando F.S., Hugo O.R., Edda L. (2007). Antioxidant activities of sulfated polysaccharides from brown and red seaweeds. J. Appl. Phycol..

[55] Pozharitskaya O.N., Shikov A.N., Faustova N.M., Obluchinskaya E.D., Kosman V.M., Vuorela H., Makarov V.G. (2018). Pharmacokinetic and tissue distribution of fucoidan from *Fucus vesiculosus* after oral administration to rats. Mar. Drugs.

[56] Lean Q.Y., Eri R.D., Fitton J.H., Patel R.P., Gueven N. (2015). Fucoidan extracts ameliorate acute colitis. PLoS ONE.

[57] Richards C., Williams N.A., Fitton J.H., Stringer D.N., Karpiniec S.S., Park A.Y. (2020). Oral fucoidan attenuates lung pathology and clinical signs in a severe influenza a mouse model. Mar. Drugs.

[58] Irhimeh M.R., Fitton J.H., Lowenthal R.M., Kongtawelert P. (2005). A quantitative method to detect fucoidan in human plasma using a novel antibody. Methods Find Exp. Clin..

[59] Tokita Y., Nakajima K., Mochida H., Iha M., Nagamine T. (2010). Development of a fucoidan-specific antibody and measurement of fucoidan in serum and urine by sandwich ELISA. Biosci. Biotechnol. Biochem..

[60] Ale M.T., Maruyama H., Tamauchi H., Mikkelsen J.D., Meyer A.S. (2011). Fucoidan from *Sargassum* sp. and *Fucus vesiculosus* reduces cell viability of lung carcinoma and melanoma cells in vitro and activates natural killer cells in mice in vivo. Int. J. Biol. Macromol..

[61] Gómez-Ordóñez E., Jiménez-Escrig A., Rupérez P. (2012). Molecular weight distribution of polysaccharides from edible seaweeds by high-performance size-exclusion chromatography (HPSEC). Talanta.

[62] OECD (2001). Guideline for Testing of Chemicals: Acute Oral Toxicity, Acute Toxic Class Method.

[63] Brooks K.J., Wiescinski C.M., Golden R. (2008). MXDE-208: Acute Oral Toxicity Study in CRL: CD1 (ICR) Mice (Up and Down Procedure).

[64] Li D.Y., Xu R.Y., Zhou W.Z., Sheng X.B., Yang A.Y., Cheng J.L. (2002). Effects of fucoidan extracted from brown seaweed on lipid peroxidation in mice. Acta Nutr. Sin..

[65] Ponnan A., Kulanthaiyesu A., Marudhamuthu M., Palanisamy K., Kadarkarai M. (2020). Protective effects of fucoidan against 4-nitroquinolin-1-oxide provoked genetic damage in mouse bone marrow cells. Environ. Sci. Pollut. Res. Int..

